# The association of the difference in hemoglobin levels before and after hemodialysis with the risk of 1-year mortality in patients undergoing hemodialysis. Results from a nationwide cohort study of the Japanese Renal Data Registry

**DOI:** 10.1371/journal.pone.0210533

**Published:** 2019-01-10

**Authors:** Hiroki Nishiwaki, Takeshi Hasegawa, Fumihiko Koiwa, Takayuki Hamano, Ikuto Masakane

**Affiliations:** 1 The Japanese Society for Dialysis Therapy, Committee of Renal Data Registry, Tokyo, Japan; 2 Division of Nephrology (Fujigaoka Hospital), Department of Medicine, Showa University School of Medicine, Yokohama, Japan; 3 Office for Promoting Medical Research, Showa University, Tokyo, Japan; 4 Department of Comprehensive Kidney Disease Research, Osaka University Graduate School of Medicine, Osaka, Japan; 5 Yabuki Hospital, Yamagata, Japan; University of Sao Paulo Medical School, BRAZIL

## Abstract

**Background:**

Few clinical studies have directly examined the associations of hemoglobin (Hb) levels after hemodialysis (HD) and of the difference in Hb levels before and after HD (ΔHb) with patient outcomes. The present study aimed to determine ΔHb and post-HD Hb levels with nationwide data and to examine their associations with all-cause mortality in patients undergoing HD.

**Methods:**

This study is based on data from 2008 and 2009 recorded in the Japanese Renal Data Registry. Study endpoints were all-cause mortality within 1-year. The ΔHb and post-HD Hb level as categorical variables using Cox regression for 1-year mortality, adjusting for potential confounders.

**Results:**

The median ΔHb was 1.0 g/dl, and the post-HD Hb level was 11.3 g/d. The median pre-HD Hb level was 10.4 g/dl. The risk of mortality was lower with a ΔHb of 0 to 1.0 g/dl (adjusted hazard ratio [aHR], 0.90; 95% confidence interval [CI], 0.70–1.01) or > 1.0 g/dl (aHR, 0.73; 95% CI, 0.64–0.84) than with a ΔHb < 0 g/dl. The risk for mortality was also lower with a post-HD Hb of 10 to 11 g/dl (aHR, 0.82; 95% CI, 0.73–0.92), 11 to 12 g/dl (aHR, 0.77; 95% CI, 0.68–0.87), or > 12 g/dl (aHR, 0.77; 95% CI, 0.68–0.87) than with a post-HD Hb < 10 g/dl.

**Conclusions:**

Both a low ΔHb and a low post-HD Hb level were associated with a higher risk of 1-year mortality.

## Introduction

The hemoglobin (Hb) level before hemodialysis (HD) is generally used as an indicator to treat renal anemia in patients undergoing HD and is also included in most clinical research studies and guidelines [1,2]. However, several studies have described post-HD Hb levels and hematocrit values (S1 Table) [3–10], and a recent study has found that Hb levels 4, 24, and 48 hours after an HD session remain as high as pre-Hb levels and do not differ significantly from immediate post-HD Hb levels [6]. By measuring post-HD Hb levels, we can obtain information about the difference in Hb levels between before and after HD (hereinafter called “ΔHb”). However, to our knowledge, only a single study [10] has examined ΔHb in detail. In addition, few clinical research studies have directly examined the association between post-HD Hb levels and patient outcomes. Therefore, the interpretation of ΔHb and post-HD Hb levels could still have been based on a physician’s clinical experience.

The aim of the present study was to describe ΔHb and post-HD Hb levels by means of data from throughout Japan and to examine the associations of ΔHb and post-HD Hb with all-cause mortality in patients undergoing HD.

## Materials and methods

### Study design, data source, and study samples

This study was designed as a prospective cohort study based on data from 2008 and 2009 recorded in the Japanese Renal Data Registry (JRDR), an HD registry of the Japanese Society of Dialysis Therapy (JSDT). The design and detailed methods of this questionnaire-based national survey have been described previously [11]. Briefly, the JSDT has conducted annual surveys of dialysis facilities throughout Japan since 1968. The response rate to the survey is > 98% each year. Because post-HD Hb was collected only during 2008, data from 2008 and 2009 were used in this study. A standard analysis file (JRDR-13107) that was prepared for this study contained 273,237 records of living Japanese patients receiving HD as of December 31, 2008. Eligible patients were those 16 years or older for whom Hb data from before and after HD was available. Exclusion criteria were (1) the outcome in 2009 being unclear; (2) the patient having undergone both HD and peritoneal dialysis at baseline; (3) the patient having been treated for the acute phase of myocardial infarction, cerebral infarction, or intracerebral hemorrhage at baseline; and (4) an HD duration < 1 year. The JRDR conducted in 2008 to 2009 followed the Ethical Guidelines for Medical and Health Research Involving Human Subjects provided in 2008 by the Japanese Ministry of Health, Labour and Welfare. On the basis of these guidelines, all patients had the opportunity to reject participation in the present study. In December 2014, the Ethical Guidelines for Medical and Health Research Involving Human Subjects, which was issued by the Ministry of Health, Labour and Welfare and the Ministry of Education, Culture, Sports, Science, and Technology, was revised and demanded that all academic societies strictly follow ethical considerations and protect personal information in epidemiological research [12]. Therefore, the present study’s protocol was reviewed and approved by the Ethics Committee of the Japanese Society of Dialysis Therapy (Approval Number 15).

### Measurement

The data included demographic information (age, sex, HD duration, cause of kidney failure, type of vascular access, and history of cardiovascular disease) and clinical data, such as pre- and post-HD Hb, blood urea nitrogen (BUN); serum levels of creatinine, sodium, potassium, chloride, inorganic phosphate, calcium (adjusted), and albumin; single-pool Kt/V; length in time of an HD session; body mass index (BMI); normalized protein catabolic rate (nPCR); and pre-HD and post-HD body weights (BW). The calcium values were corrected for the serum albumin concentration [13]. The BW loss during the hemodialysis session divided by the pre-dialysis BW was defined as %ΔBW. All laboratory data were collected at the first HD session of a week. The information of mortality included the cause and date (year and month) of death.

The primary exposures were ΔHb, which were defined as post-HD Hb levels minus pre-HD Hb levels. The ΔHb was used as an ordinal variable, which was categorized as < 0, 0 to < 1 and ≥ 1 g/dl. The post-HD Hb level was also used as a continuous variable and an ordinal variable. Post-HD Hb levels were categorized into 4 groups and used as ordinal variables: < 10, ≥ 10 to < 11, ≥ 11 to < 12, and ≥ 12 g/dl.

As the primary outcome of this study, all-cause mortality during the study period was defined.

### Statistical analysis

Baseline data of the study patients were divided into 3 groups based on baseline characteristics of ΔHb; for all patients and these groups the continuous variables are presented as median values and quartiles, and the categorical variables are presented as numbers and percentages. Comparisons of continuous variables among the groups were assessed with the analysis of variance (ANOVA), and comparisons of categorical data were assessed with the Chi-square test. The distribution of ΔHb and the post-HD and pre-HD Hb levels are presented with histograms by means of kernel density estimation. In addition, the proportion of missing values was calculated with the number of patients meeting eligibility and exclusion criteria as the denominator. As supplementary material, the proportion of missing values for patients whose post-HD Hb levels were not measured was calculated with the number of samples meeting only exclusion criteria as the denominator.

Missing values of covariates were multiply imputed in primary analysis assuming that missing values were at random. To impute the missing values we constructed multiple regression models including variables potentially related to the fact that the data were missing and variables correlated with that outcome. The results across 20 imputed data sets were combined by averaging, and standard errors were adjusted to reflect both within-imputation and between-imputation variability. These estimates and their standard errors were combined by means of Rubin’s rules.

The variables affecting ΔHb were examined with a multivariable logistic regression model. In consideration of multicollinearity, the correlation coefficient of each candidate continuous variable was calculated before logistic regression analysis. When the correlation coefficient was –1 to –0.7 or was 0.7 to 1.0, these variables were eliminated from the model. Multiple stepwise analysis was then performed to identify significant independent effects on ΔHb. The candidate variables were age, sex, HD duration, cause of end-stage renal disease (ESRD), history of cardiovascular disease or amputation, intradialysis BW loss, BMI, serum albumin level, length of time of HD session, BUN, serum creatinine, serum sodium, serum potassium, adjusted calcium, phosphate, PCR, Kt/V, and the type of blood access.

For the association between each ΔHb and outcomes, Cox proportional hazard analysis was performed for 1-year mortality. Cox proportional hazard analysis was also performed with post-HD Hb as exposure.

All models were adjusted for the full variables; age, sex, HD duration, cause of ESRD, history of cardiovascular disease or amputation, intradialysis BW loss, BMI, serum albumin level, time length of HD session, BUN, serum creatinine, serum sodium, serum potassium, adjusted calcium, phosphate, PCR, Kt/V, C-reactive protein (CRP), and the type of blood access. In consideration of the non-normal distribution, CRP was used as a variable after logarithmic transformation. Because pre- and post-HD Hb levels were expected to be correlated, both were not included in the same multivariable model [14]. Subgroup analysis was stratified with pre-HD Hb groups categorized to < 10, ≥ 10 to ≤ 12, and >12 g/dl on the basis of guidelines for renal anemia [1]. In consideration of the fluid removal affecting ΔHb, the analysis was increased in the following ways. First, stratified analysis was performed with binary data of ΔBW to examine the association between ΔHb and mortality. Second, analysis was performed of the interaction with ΔHb and ΔBW as dichotomous variables.

Sensitivity analysis was performed with tertiles of ΔHb and quartiles of post-HD Hb levels.

On the basis of a previous study of pre-HD Hb levels [15], the association between post-HD Hb levels and the outcome was expected to be nonlinear. Accordingly, the association is shown with the restricted cubic spline method and the 4 knots of the 20th, 40th, 60th, and 80th percentile points of each Hb level. The association is presented as linear for less than 20 percentile points and more than 80 percentile points and as a restricted cubic spline from the 20 to 80 percentiles.

To confirm the direction of selection bias, the chi-squire test was used for the mortality between patients for whom post-HD Hb was or was not measured.

All analyses were performed with the statistical software programs Stata 14.2 (StataCorp, College Station, TX, USA) and R version 3.4.1 (The R Foundation for Statistical Computing, https://www.r-project.org) and 2-sided significance set at 0.05. Hazard ratios were estimated for all-cause with their 95% confidence intervals.

## Results

### Baseline characteristics

**Fig 1:**
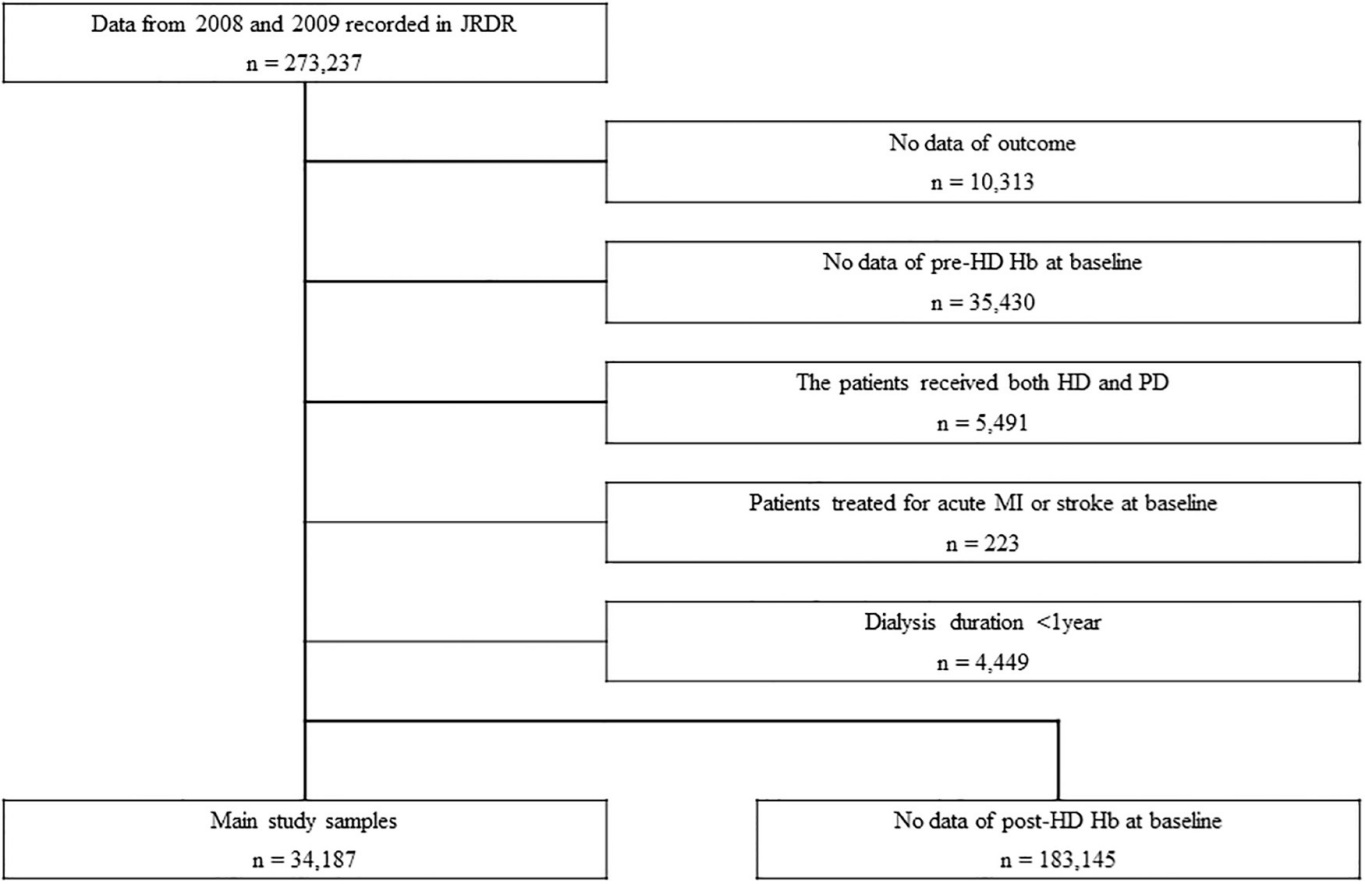
Study profile. JRDR, Japanese Renal Data Registry; Hb, hemoglobin levels; HD, hemodialysis; PD, peritoneal dialysis; MI, myocardial infarction.

Eligible subjects were 34,187 patients (Fig 1). The subjects had a median age of 66 years and a median HD duration of 6 years; 38.6% of subjects were women, 89.9% had an arteriovenous fistula for vascular access, and 24.5% had a history of cardiovascular disease. The most common cause of kidney failure was glomerulonephritis (Table 1).

**Table 1:** Patient characteristics.

	All				Categorized by ΔHb (g/dl)						
	n = 34,187		Missing		< 0		0 to ≤ 1		> 1		
			n	(%)	n = 3,393 (%)		n = 13,699 (%)		n = 17,095 (%)		p-value
Age, years (Q1, Q3)	66	(57, 74)	0	(0.0%)	68	(60, 76)	67	(59, 75)	64	(55, 72)	<0.01
Sex, female, %	13,178	(38.6%)	0	(0.0%)	1,200	(35.4%)	5,298	(38.7%)	6,680	(39.1%)	<0.01
Duration of dialysis, median years (Q1, Q3)	6	(3,11)	2	(0.0%)	5	(2, 11)	6	(3,10)	6	(3, 11)	0.06
Cause of ESRD, n (%)			0	(0.0%)							<0.01
Glomerulonephritis	14,507	(42.4%)			1,342	(39.6%)	5,417	(39.5%)	7,748	(45.3%)	
Diabetic nephropathy	11,391	(33.3%)			1,179	(34.8%)	5,033	(36.7%)	5,179	(30.3%)	
Nephrosclerosis	2,200	(6.4%)			231	(6.8%)	908	(6.6%)	1,061	(6.2%)	
PKD	1,138	(3.3%)			142	(4.2%)	499	(3.6%)	497	(2.9%)	
RPGN	225	(0.7%)			23	(0.7%)	65	(0.5%)	137	(0.8%)	
Others	2,407	(7.0%)			240	(7.1%)	846	(6.2%)	1,321	(7.7%)	
Unknown	2,319	(6.8%)			236	(7.0%)	931	(6.8%)	1,152	(6.7%)	
Vascular access			1,781	(5.2%)							0.053
AVF	29,130	(89.9%)			2,847	(89.5%)	11,605	(89.5%)	14,678	(90.3%)	
AVG	2,420	(7.5%)			244	(7.7%)	992	(7.7%)	1,184	(7.3%)	
Others	856	(2.6%)			91	(2.9%)	375	(2.9%)	390	(2.4%)	
Length of HD session, hours	4	(4, 4)	22	(0.1%)	4	(3.5, 4)	4	(4, 4)	4	(4, 4)	<0.01
Comorbidities											
Cardiovascular disease, %	7,041	(24.5%)	5,458	(16.0%)	2,158	(75.0%)	8,547	(73.8%)	10,983	(77.0%)	<0.01
Amputation, %	857	(3.0%)	5,445	(15.9%)	88	(3.1%)	360	(3.1%)	409	(2.9%)	0.47

HD, hemodialysis; Hb, hemoglobin; ΔHb, difference in Hb levels before and after HD; Q1, first quartile; Q3, third quartile; ESRD, end-stage renal disease; PKD, polycystic kidney disease; RPGN, rapid progressive glomerulonephritis; AVF, arteriovenous fistula; AVG, arteriovenous graft. Age and duration of dialysis is presented with median (Q1, Q3). All categorical values are presented with n (%). Denominator of missing variables is the number of all patients (n = 34,187).

**Fig 2:**
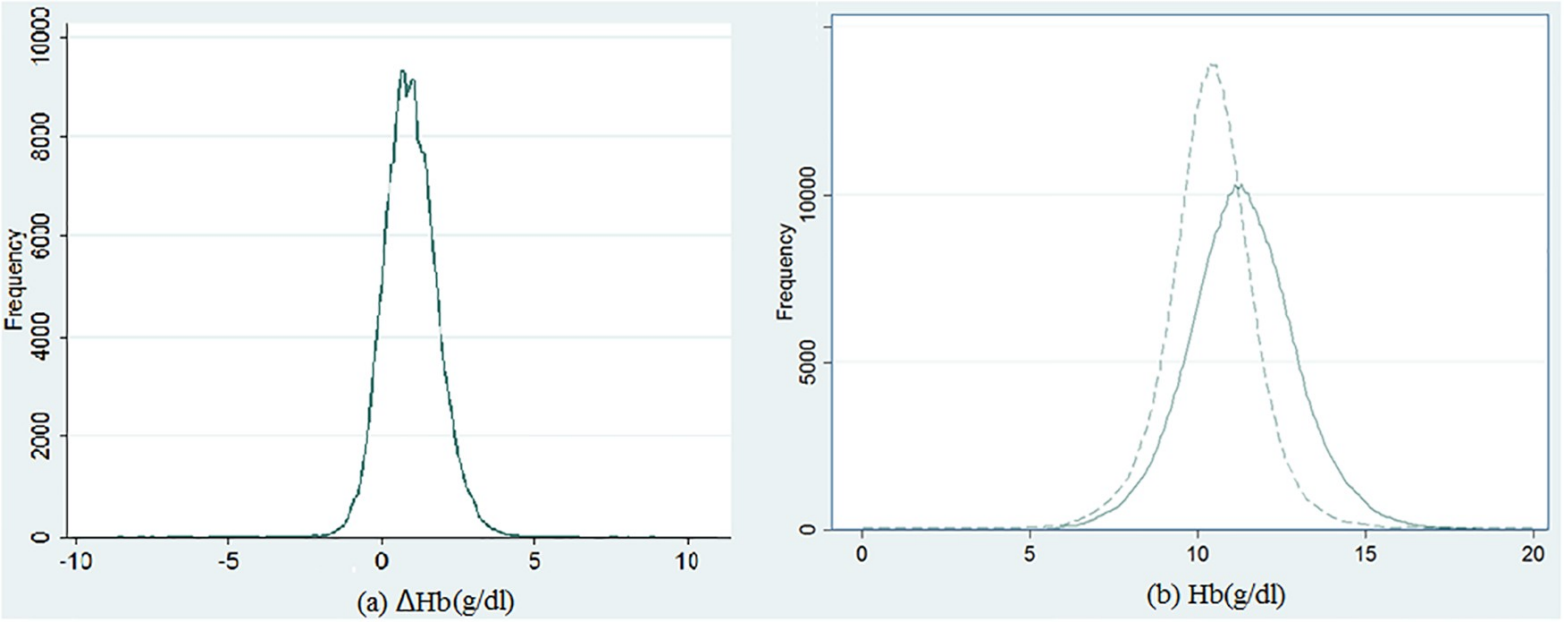
Histogram with kernel density estimation of (a) difference in hemoglobin levels before and after hemodialysis (ΔHb), and (b) pre-hemodialysis and post-hemodialysis hemoglobin. Solid line presents post-hemodialysis hemoglobin, and dashed line presents pre-hemodialysis hemoglobin.

The median values were pre-HD Hb level, 10.4 g/dl; post-HD Hb level, 11.3 g/dl; and ΔHb, 0.9 g/dl (Fig 2). Patients with a higher ΔHb were younger and had a higher proportion of glomerulonephritis and a lower proportion of diabetes nephropathy as causes of ESRD, a higher proportion of arteriovenous fistula, and a lower proportion of amputation. Patients with a higher ΔHb had higher levels of creatinine, BUN, potassium, inorganic phosphate, Kt/V, nPCR, and intradialytic BW loss (Tables 1 and 2). The patient’s characteristics and laboratory data stratified by post-hemodialysis hemoglobin were in S2 and S3 Tables.

**Table 2:** Laboratory data.

	All				Categorized by ΔHb (g/dl)						
	n = 34,187		Missing		< 0		≥ 0 to ≤ 1		> 1		
			n	(%)	n = 3,393 (9.9%)		n = 13,669 (40.0%)		n = 17,095 (50.0%)		p-value
ΔHb, g/dl	1.0	(0.4, 1.5)	0	(0.0%)	-0.3	(-0.6, -0.2)	0.5	(0.3, 0.7)	1.5	(1.2, 2.0)	<0.01
Post-HD Hb, mean g/dl	11.3	(10.3, 12.4)	0	(0.0%)	9.9	(9.1, 10.7)	10.7	(9.9, 11.5)	12.1	(11.3, 13.1)	<0.01
Pre-HD Hb, mean g/dl	10.4	(9.6, 11.1)	0	(0.0%)	10.3	(9.5, 11.2)	10.2	(9.5, 11.0)	10.5	(9.8, 11.3)	<0.01
Pre-HD serum albumin, mg/dl	3.7	(3.5 4.0)	611	(1.8%)	3.8	(3.4, 4.0)	3.7	(3.5, 4.0)	3.7	(3.5, 3.9)	<0.01
Pre-HD BUN, mg/dl	64	(54, 75)	9	(0.0%)	56	(46, 67)	62	(51, 72)	67	(57, 78)	<0.01
Pre-HD serum creatinine, mg/dl	10.5	(8.6, 12.4)	11	(0.0%)	9.1	(7.5, 10.9)	10	(8.3, 11.8)	11.2	(9.3, 13.0)	<0.01
Pre-HD sodium, mEq/L	139	(137, 141)	21	(0.1%)	139	(137, 141)	139	(137, 141)	139	(137, 141)	<0.01
Pre-HD potassium, mEq/L	5.0	(4.4, 5.5)	21	(0.1%)	4.7	(4.1, 5.2)	4.9	(4.4, 5.4)	5.1	(4.6, 5.6)	<0.01
Pre-HD calcium (adjusted), mg/dl	9.3	(8.8, 9.9)	676	(2.0%)	9.4	(8.9, 9.9)	9.3	(8.9, 9.9)	9.3	(8.8, 9.8)	<0.01
Pre-HD phosphate, mg/dl	5.2	(4.3, 6.1)	131	(0.4%)	4.7	(3.9, 5.6)	5	(4.2, 6.0)	5.4	(4.6, 6.4)	<0.01
CRP	0.11	(0.05, 0.38)	4019	(11.8%)	0.15	(0.06, 0.53)	0.11	(0.05, 0.40)	0.11	(0.05, 0.31)	<0.01
Kt/V, ml/min	1.4	(1.22, 1.58)	305	(0.9%)	1.31	(1.11, 1.51)	1.37	(1.20, 1.55)	1.42	(1.25, 1.62)	<0.01
nPCR, g	0.9	(0.76, 0.99)	259	(0.8%)	0.76	(0.65, 0.88)	0.84	(0.73, 0.96)	0.92	(0.81, 1.04)	<0.01
BMI, kg/m^2^	20.8	(18.8, 23.1)	4555	(13.3%)	20	(18.2, 22.2)	20.5	(18.6, 22.7)	21.2	(19.1, 23.6)	<0.01
%ΔBW, %	4.6	(3.5, 5.7)	206	(0.5%)	3.1	(1.7, 4.4)	4.2	(3.2, 5.3)	5.1	(4.2, 6.1)	<0.01

HD, hemodialysis; Hb, hemoglobin; ΔHb, difference in Hb levels before and after HD; BUN, blood urea nitrogen; BW, body weight; CRP, C-reactive protein; nPCR, normalized protein catabolic rate; BMI, body mass index. All variables are presented as median (1st quartile, 3rd quartile). Denominator of missing variables is the number of all patients (n = 34,187).

### Clinical factors associated with higher ΔHb

The correlation coefficient calculated for Kt/V and nPCR was 0.70 and that for % ΔBW and PCR was 0.85 (S4 Table). Therefore, nPCR and Kt/V were excluded from candidate variables affecting ΔHb. Multivariable regression analysis with stepwise analysis for ΔHb showed that the factors negatively related with ΔHb were age, male sex, HD duration, the levels of serum sodium and serum albumin, and the adjusted serum levels of calcium and CRP. The factors positively related with ΔHb were female sex, history of cardiovascular disease, BMI, % ΔBW, and the levels of BUN, serum creatinine, and serum potassium (Table 3). Multiple stepwise analysis showed that the factors related with ΔHb were age, sex, HD vintage, ΔBW, BMI, and the levels of CRP, BUN, creatinine, Na, K, albumin, and calcium.

**Table 3:** The factors related with difference in hemoglobin levels before and after hemodialysis > 0.

	multivariable analysis				Stepwise multivariable analysis			
	Odds Ratio	95% confidence interval		p-value	Odds Ratio	95% confidence interval		p-value
Age (5-year increment)	0.98	0.96	1.00	0.06	0.95	0.92	0.98	<0.01
Sex (male)	0.64	0.59	0.70	<0.01	0.57	0.48	0.67	<0.01
HD vintage (1-year increment)	0.98	0.98	0.99	<0.01	0.98	0.97	0.99	<0.01
Times of HD session (60-min increment)	1.00	0.89	1.12	0.98				
CVD	1.23	1.11	1.36	<0.01				
Amputation	1.10	0.86	1.42	0.45				
Cause of ESRD								
Glomerulonephritis	Reference							
Diabetic nephropathy	0.91	0.82	1.01	0.08				
Nephrosclerosis	1.01	0.85	1.20	0.87				
PKD	0.62	0.50	0.76	<0.01				
RPGN	0.84	0.52	1.36	0.47				
Others	0.90	0.76	1.07	0.25				
Unknown	0.99	0.84	1.17	0.92				
Vascular access								
AVF	Reference							
AVG	1.10	0.94	1.28	0.24				
Others	1.14	0.89	1.46	0.31				
Pre-HD albumin	0.42	0.37	0.47	<0.01	0.43	0.35	0.52	<0.01
Pre-HD BUN (10 mg/dl increment)	1.24	1.20	1.28	<0.01	1.31	1.23	1.38	<0.01
Pre-HD creatinine	1.16	1.14	1.19	<0.01	1.17	1.13	1.21	<0.01
Pre-HD sodium (5 mEq/L increment)	0.93	0.87	1.00	0.07	0.81	0.72	0.92	<0.01
Pre-HD potassium	1.10	1.03	1.17	0.01	1.21	1.08	1.35	<0.01
Pre-HD calcium (adjusted)	0.82	0.78	0.87	<0.01	0.82	0.74	0.90	<0.01
Pre-HD phosphate	1.03	0.99	1.07	0.10				
CRP	0.94	0.92	0.96	<0.01	0.94	0.91	0.98	<0.01
BMI	1.09	1.07	1.10	<0.01	1.05	1.02	1.05	<0.01
%ΔBW (1% increment)	1.31	1.17	1.46	<0.01	1.04	1.02	1.05	<0.01

HD, hemodialysis; Hb, hemoglobin; CI: confidence interval; CVD, cardiovascular disease; ESRD, end-stage renal disease, PKD: polycystic kidney disease; RPGN, rapid progressive glomerulonephritis; AVF, arteriovenous fistula; AVG, arteriovenous graft; BUN, blood urea nitrogen; CRP, C-reactive protein; BMI, body mass index; BW, body weight; Statistical significance set at 0.05.

### The association between exposures and 1-year mortality

**Fig 3:**
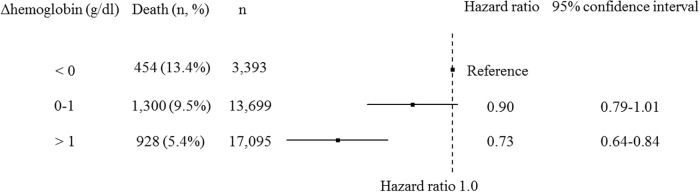
The association between the categorized difference in hemoglobin levels before and after hemodialysis (ΔHb) of all patients and all-cause mortality (1-year) in the Cox proportional hazard model. The model was adjusted with age, sex, dialysis duration, cause of end-stage renal disease, history of cardiovascular disease or amputation, body mass index, serum albumin level, time of dialysis session, blood urea nitrogen, serum creatinine, serum sodium, serum potassium, adjusted calcium, phosphate, C-reactive protein, protein catabolic rate, Kt/V, the amount of fluid removal, and type of blood access.

**Fig 4:**
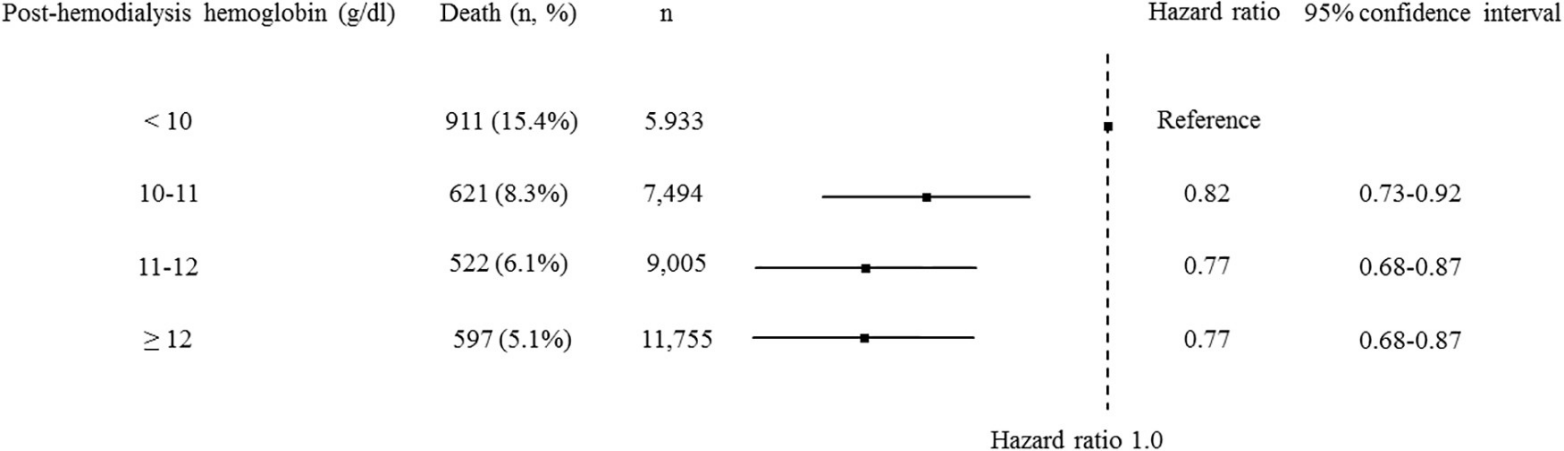
The association between categorized post-hemodialysis (HD) hemoglobin and all-cause mortality (1-year) in the Cox proportional hazard model. The model was adjusted with age, sex, HD duration, cause of end-stage renal disease, history of cardiovascular disease or amputation, body mass index, serum albumin level, time of HD session, blood urea nitrogen, serum creatinine, serum sodium, serum potassium, adjusted calcium, phosphate, C-reactive protein, protein catabolic rate, Kt/V, the amount of fluid removal, and type of blood access.

**Fig 5:**
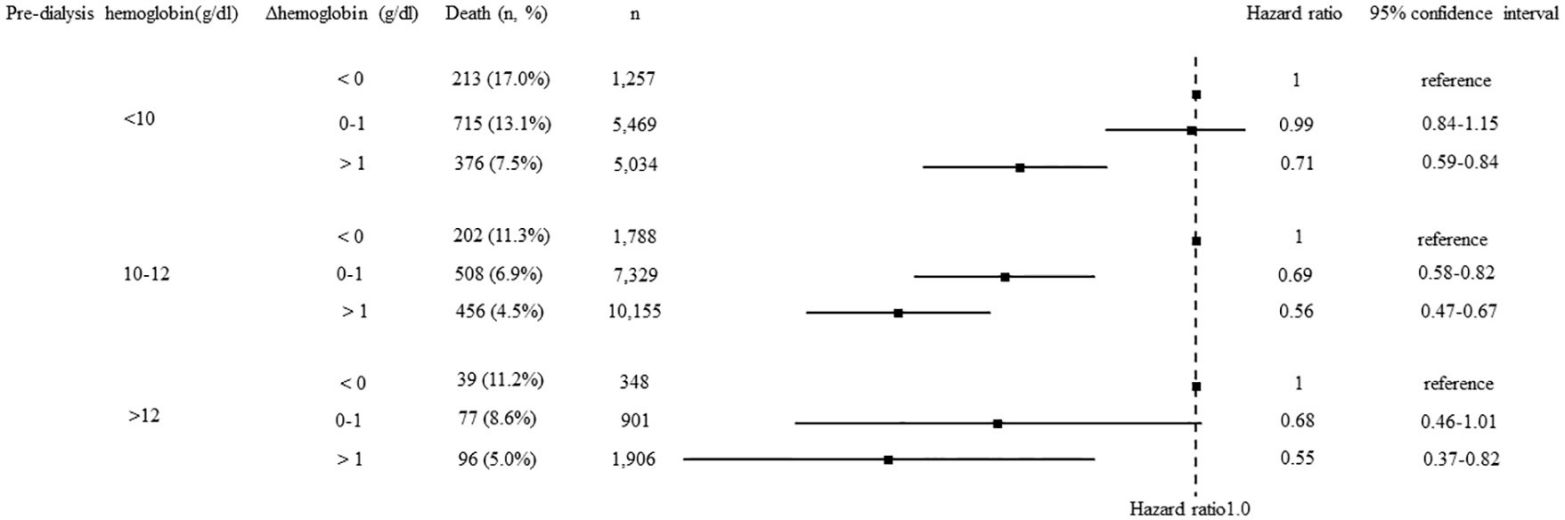
The association between the difference in hemoglobin levels before and after hemodialysis (ΔHb) stratified with the pre-hemodialysis (HD) hemoglobin level and all-cause mortality. The model was adjusted with age, sex, HD duration, cause of end-stage renal disease, history of cardiovascular disease or amputation, body mass index, serum albumin level, time of HD session, blood urea nitrogen, serum creatinine, serum sodium, serum potassium, adjusted calcium, phosphate, C-reactive protein, protein catabolic rate, Kt/V, the amount of fluid removal, and type of blood access.

During the 1-year follow-up period, accounting for 482,179 person-months of follow-up, all-cause deaths occurred for 2,682 patients. During the 1-year follow-up period, the cases of 108 patients were censored, because of discontinuation of HD for any reason in 90 patients and because of being recipients of kidney transplantation in 18 patients. A lower ΔHb was related with a higher 1-year mortality (Fig 3). The post-HD Hb level also had a significant association with all-cause mortality in an adjusted Cox proportional hazard model (Fig 4). The association of ΔHb stratified pre-HD Hb level with all-cause mortality showed that each stratum presented similar trends (Fig 5). A similar trend was found with sensitivity analysis using quartiles of ΔHb levels (S1 Fig). In contrast, morality was lower with the highest quartile of post-HD Hb levels than with the third quartile. In consideration of interaction, similar analysis was performed with ΔBW as the stratum. A similar trend was found (S2 Fig), but the interaction between % ΔBW and ΔHb was not statistically significant.

The post-HD Hb levels were measured in 38,670 patients, of whom 3,068 (7.94%) died, and were not measured in 184,568 patients, of whom 14,858 (8.05%) died; the percentage of deaths did not differ significantly between these patient groups (p = 0.46). The baseline characteristics and laboratory data were similar between patients for whom the post-HD Hb level was or was not measured (S5 and S6 Tables). Missing data was more common among patients for whom the post-HD Hb level was not measured.

## Discussion

In the present study, the post-HD Hb level was measured in 17% of patients in the 2008–2009 JRDR database. The median ΔHb was 0.9 g/dl, and the mean post-HD Hb level was 11.3 g/dl. To our knowledge, the present study has described the largest collection of data on ΔHb and post-HD Hb.

Previous studies have found that ΔHb is mainly affected by the amount of fluid removed during HD [3,4,8]. Therefore, these studies suggest that ΔHb < 0 g/dl is related to insufficient intradialysis fluid removal and dilution during the HD session. The present study has shown that lower ΔHb is related to age, sex, HD duration, and serum levels of albumin, sodium, calcium, and CRP. In contrast, with higher serum levels of BUN, creatinine and potassium, ΔHb was increased. Therefore, we believe that a favorable nutritional status might be related with ΔHb > 0 g/dl regardless of the HD duration. However, the serum albumin level showed an inverse correlation with ΔHb. Because our analysis was cross-sectional, the results can be retrocausal.

The present study has shown that lower ΔHb is related to a higher 1-year risk of mortality. The result is possibly robust, because this trend was found by means of the analysis stratified with the pre-HD Hb level or sensitivity analysis. Furthermore, our study showed a dose-response relationship between ΔHb and 1-year mortality. In sub-analysis, some patient groups did not show results with statistical significance; however, these results are affected by sample size and effect size. Patients with pre-HD Hb level < 10 g/dl also did not clearly show a dose-dependent response relationship between ΔHb and mortality. Our finding that ΔHb was not related to mortality in the group of pre-HD Hb <10 might be due to a possible relationship reported between a pre-HD Hb level < 10 g/dl and higher mortality [15,16]. Crude mortality with pre-HD Hb < 10 g/dl in the present study was consistent with these previous studies. Therefore, our study suggests that clinicians should first achieve a pre-HD Hb level within a target range and then improve ΔHb.

Our study has also shown that a lower post-HD Hb level is associated with a high 1-year mortality rate. This relation to the mortality rate was similar to that reported for pre-HD Hb levels [15]. In addition, we found in our primary analysis that a higher post-HD Hb level was related to a lower mortality rate (Fig 4). In sensitivity analysis, the hazard ratio of the 4^th^ quartile of post-HD Hb was slightly lower than that of the 3^rd^ quartile and suggested a U-shaped curve relationship (S3 Fig). Therefore, to analyze the relationship between post-HD Hb and 1-year mortality, we performed logistic analysis with a restricted cubic spline. This analysis showed a U-shaped curve (S4 Fig). Previous observational studies have suggested that higher Hb levels do not increase the mortality rate [15,16]; however, randomized control trials have shown that high Hb levels worsen clinical outcomes by increasing the risks of stroke, hypertension, and vascular access thrombosis [17–20] but do not increase the mortality rate. In contrast, our present study suggests that a higher ΔHb is related to a lower mortality rate and that a higher post-HD Hb level might be associated with a lower mortality rate; however, depending on analysis, the relationship between the post-HD Hb level and mortality assumed a U-shaped curve, meaning that an extremely high post-HD Hb level would greatly increase the mortality rate. Our present study suggests that the upper limit of the post-HD Hb level is 13 to 14 g/dl; however, further research is needed to determine the upper limit.

The present study has two strong points. First, this study was a nationwide study in Japan and had the largest number of subjects among studies focused on ΔHb and post-HD Hb. Second, the results were adjusted for potential confounders, including markers of inflammation.

However, the present study also has several limitations. First, the subjects of this study were only Japanese patients, who interfere with the results being generalized to a global population. A characteristic of Japanese patients with HD is a high degree of intradialytic weight gain [21]. Additionally, the subjects were only patients in whom both pre-HD and post-HD Hb levels were measured. However, we also examined mortality and baseline characteristics and found no significant difference within measured variables regardless of whether post-HD Hb levels were measured. A second limitation is that serum post-HD Hb levels and other laboratory data were measured only at baseline; therefore, the effect of changes from baseline during follow-up could not be determined with time-dependent analyses. Additionally, the follow-up period of 1 year was short. A long-term follow-up study should eventually be performed. A third limitation of the present study is that information was not collected about blood pressure, erythropoiesis-stimulating agents, iron administration, ferritin, and antihypertensive agents. In contrast, a previous study using 2005–2006 JRDR data found an association between erythropoiesis responsiveness and mortality [22]. Considering this data, our study might have underestimated the risk of mortality in patients with lower Hb levels. Another study using JRDR data showed that patients with a higher ferritin level had a higher risk of mortality than did patients with a lower ferritin level; however, transferrin saturation had no association with mortality [23]. Similarly, in yet another study using JRDR data the association between Hb levels and ferritin assumed an inversed J-shaped curve [24]. Another study showed that serum ferritin levels of 200 to 1200 ng/ml (reference, 100 to 199 ng/ml) were associated with the lowest all-cause mortality rate and that serum ferritin levels greater than 1200 ng/ml were associated with a higher all-cause mortality rate [25]. Because ferritin had a nonlinear association with Hb in the previous study and was not measured in our cohort, how our results might have been affected by ferritin is difficult to determine.

In conclusion, we found in the present study that lower ΔHb is associated with a significantly elevated 1-year risk of mortality. Lower post-HD Hb levels are also related with 1-year mortality.

## Supporting information

S1 Fig Tertile ΔHb and 1-year mortality. The association between the categorized difference in hemoglobin levels before and after hemodialysis (ΔHb) (tertile) of all patients and all-cause mortality (1-year) in the Cox proportional hazard model for sensitivity analysis. The model was adjusted with age, sex, dialysis duration, cause of end-stage renal disease, history of cardiovascular disease or amputation, body mass index, serum albumin level, time of hemodialysis session, blood urea nitrogen, serum creatinine, serum sodium, serum potassium, adjusted calcium, phosphate, C-reactive protein, protein catabolic rate, Kt/V, the amount of fluid removal, and type of blood access.
(TIF)

S2 Fig ΔHb and 1-year mortality stratified by dichotomic %ΔBW. The association between the categorized difference in hemoglobin levels before and after hemodialysis (ΔHb) of all patients and all-cause mortality (1-year) in the Cox proportional hazard model, stratified by dichotomic %ΔBW. The model was adjusted with age, sex, dialysis duration, cause of end-stage renal disease, history of cardiovascular disease or amputation, body mass index, serum albumin level, time of dialysis session, blood urea nitrogen, serum creatinine, serum sodium, serum potassium, adjusted calcium, phosphate, C-reactive protein, protein catabolic rate, Kt/V, and type of blood access.
(TIF)

S3 Fig Quartile post-hemodialysis Hb and 1-year mortality. The association between quartile post-hemodialysis hemoglobin and all-cause mortality (1-year) in the Cox proportional hazard model. The model was adjusted with age, sex, hemodialysis duration, cause of end stage renal disease, history of cardiovascular disease or amputation, body mass index, serum albumin level, time of hemodialysis session, blood urea nitrogen, serum creatinine, serum sodium, serum potassium, adjusted calcium, phosphate, C-reactive protein, protein catabolic rate, Kt/V, the amount of fluid removal, and type of vascular access.
(TIF)

S4 Fig Post-dialysis hemoglobin as continuous variable and 1-year mortality. The association between post-hemodialysis hemoglobin as continuous variable and all-cause mortality (1-year) in logistic regression analysis with a restricted cubic spline. The histogram presents the frequency of post-hemodialysis hemoglobin.
(TIF)

S1 Table Summary of previous studies dealing with post-hemodialysis hemoglobin. (DOCX)

S2 Table Patient characteristics stratified by post-hemodialysis hemoglobin. (DOCX)

S3 Table Laboratory data stratified by post-hemodialysis hemoglobin. (DOCX)

S4 Table Correlation coefficients of each candidate confounding variables. (DOCX)

S5 Table Patient characteristics stratified by measuring or not measuring post-hemodialysis hemoglobin. (DOCX)

S6 Table Laboratory data stratified by measuring or not measuring post-hemodialysis hemoglobin. (DOCX)
