# Supplementary figures and images for: The association of the difference in hemoglobin levels before and after hemodialysis with the risk of 1-year mortality in patients undergoing hemodialysis. Results from a nationwide cohort study of the Japanese Renal Data Registry
Source: PLoS One. 2019 Jan 10;14(1):e0210533. doi: 10.1371/journal.pone.0210533 (PMC6328160; doi:10.1371/journal.pone.0210533)

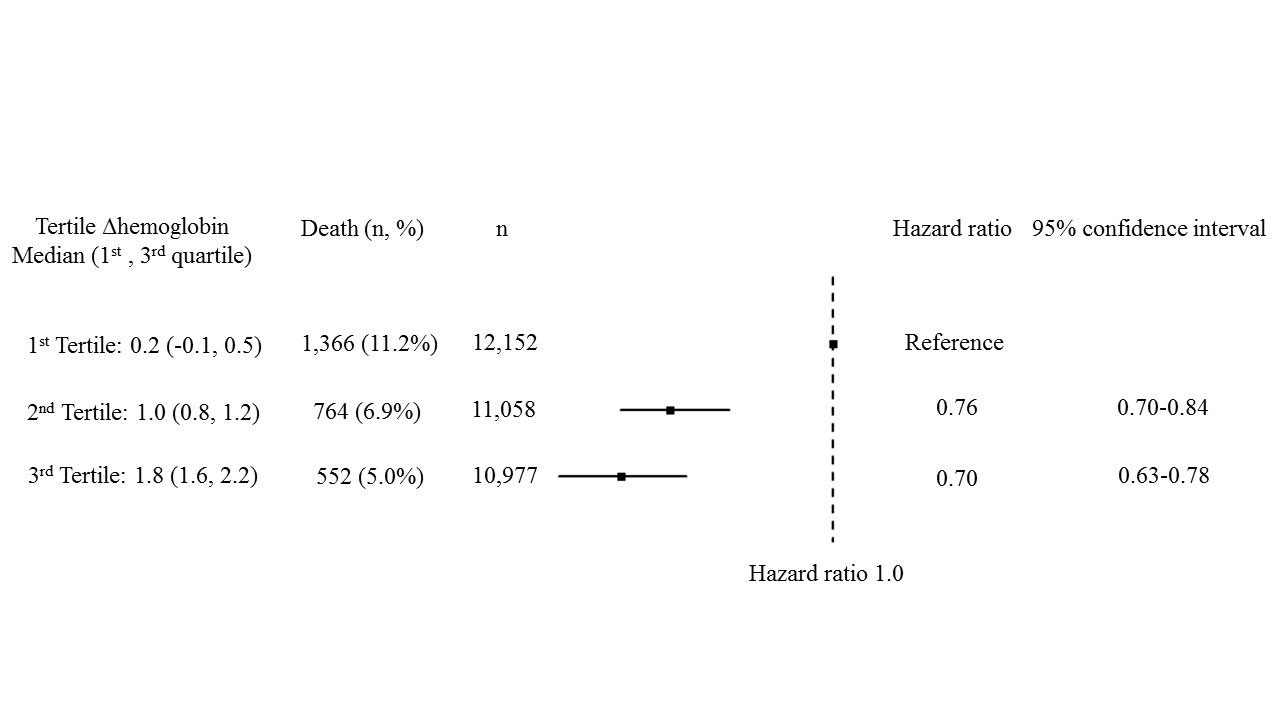

Supplement: S1 Fig — The association between the categorized difference in hemoglobin levels before and after hemodialysis (ΔHb) (tertile) of all patients and all-cause mortality (1-year) in the Cox proportional hazard model for sensitivity analysis. The model was adjusted with age, sex, dialysis duration, cause of end-stage renal disease, history of cardiovascular disease or amputation, body mass index, serum albumin level, time of hemodialysis session, blood urea nitrogen, serum creatinine, serum sodium, serum potassium, adjusted calcium, phosphate, C-reactive protein, protein catabolic rate, Kt/V, the amount of fluid removal, and type of blood access. (TIF) [file pone.0210533.s001.tif]

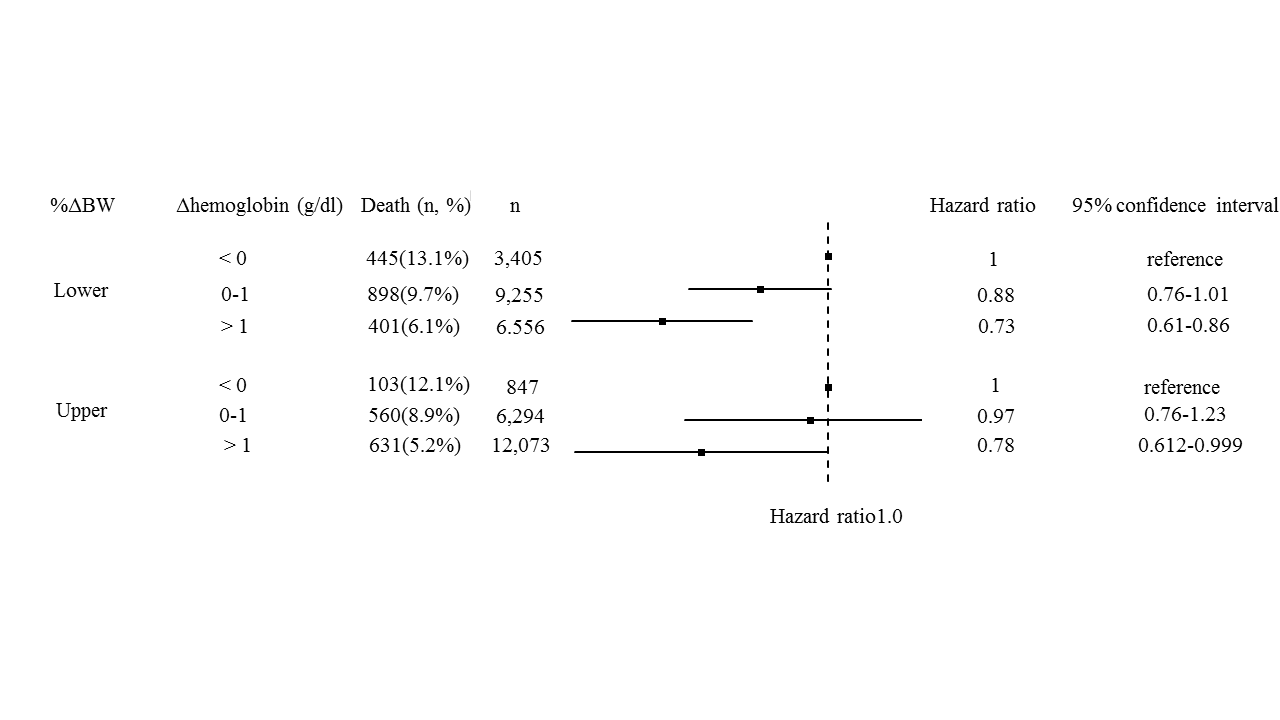

Supplement: S2 Fig — The association between the categorized difference in hemoglobin levels before and after hemodialysis (ΔHb) of all patients and all-cause mortality (1-year) in the Cox proportional hazard model, stratified by dichotomic %ΔBW. The model was adjusted with age, sex, dialysis duration, cause of end-stage renal disease, history of cardiovascular disease or amputation, body mass index, serum albumin level, time of dialysis session, blood urea nitrogen, serum creatinine, serum sodium, serum potassium, adjusted calcium, phosphate, C-reactive protein, protein catabolic rate, Kt/V, and type of blood access. (TIF) [file pone.0210533.s002.tif]

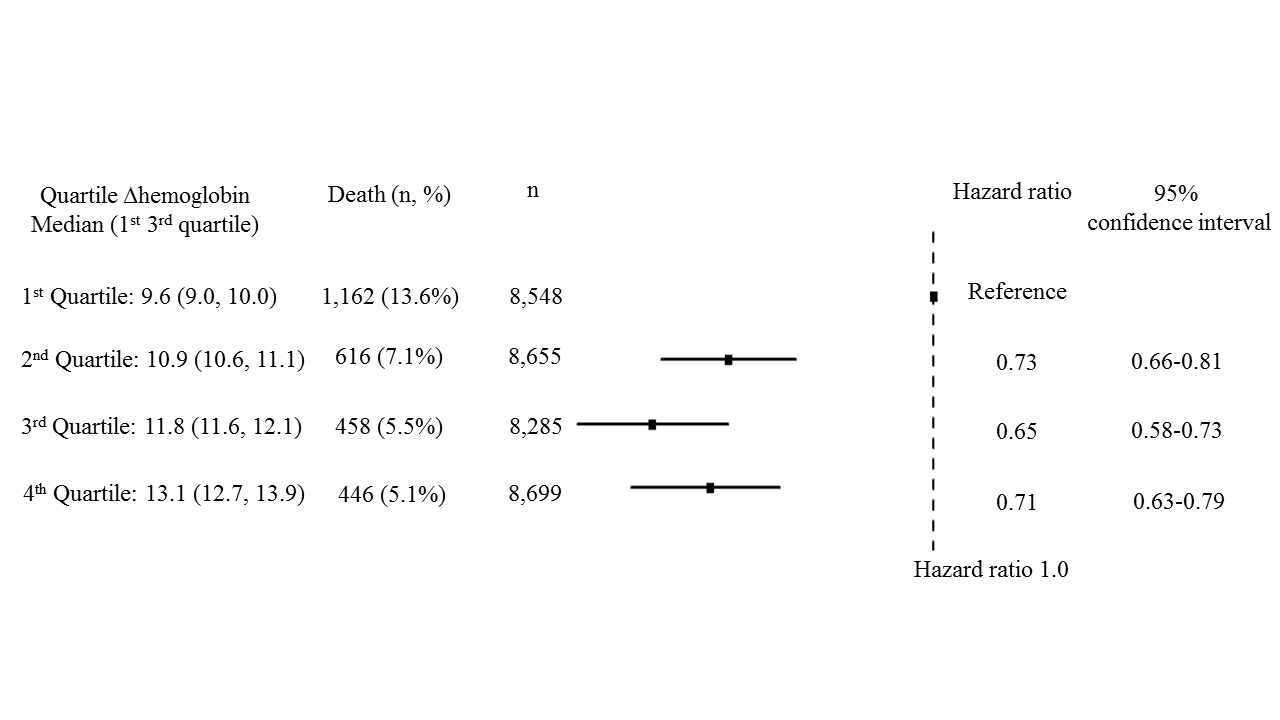

Supplement: S3 Fig — The association between quartile post-hemodialysis hemoglobin and all-cause mortality (1-year) in the Cox proportional hazard model. The model was adjusted with age, sex, hemodialysis duration, cause of end stage renal disease, history of cardiovascular disease or amputation, body mass index, serum albumin level, time of hemodialysis session, blood urea nitrogen, serum creatinine, serum sodium, serum potassium, adjusted calcium, phosphate, C-reactive protein, protein catabolic rate, Kt/V, the amount of fluid removal, and type of vascular access. (TIF) [file pone.0210533.s003.tif]

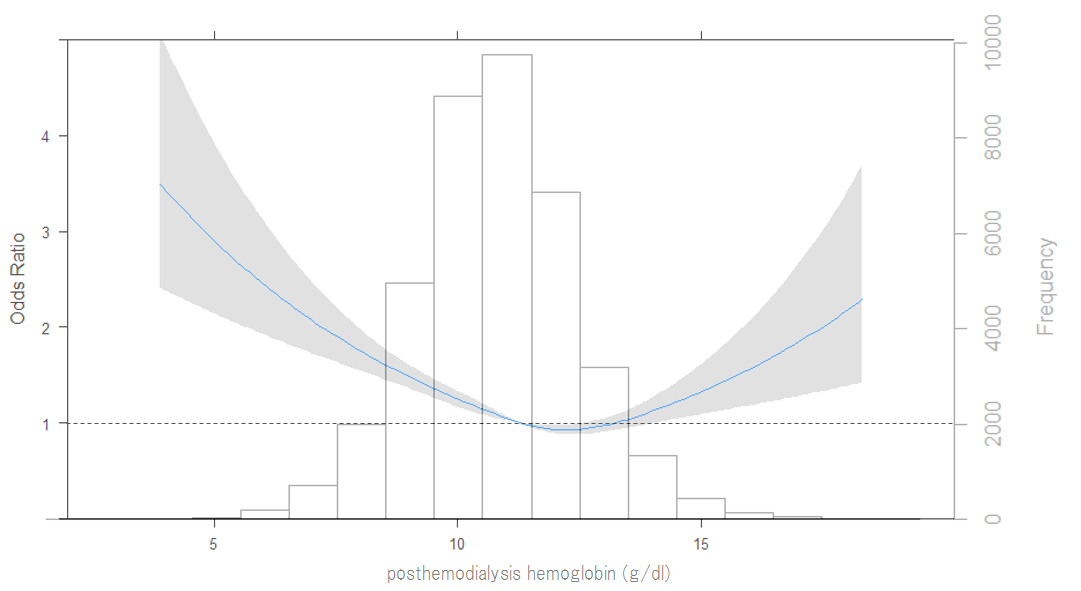

Supplement: S4 Fig — The association between post-hemodialysis hemoglobin as continuous variable and all-cause mortality (1-year) in logistic regression analysis with a restricted cubic spline. The histogram presents the frequency of post-hemodialysis hemoglobin. (TIF) [file pone.0210533.s004.tif]
