# Supplementary material for: The association of the difference in hemoglobin levels before and after hemodialysis with the risk of 1-year mortality in patients undergoing hemodialysis. Results from a nationwide cohort study of the Japanese Renal Data Registry
Source: PLoS One. 2019 Jan 10;14(1):e0210533. doi: 10.1371/journal.pone.0210533 (PMC6328160; doi:10.1371/journal.pone.0210533)
Supplement: S1 Table — (DOCX) [file pone.0210533.s005.docx]

S1 Table: Summary of previous studies dealing with post-hemodialysis hemoglobin

| Author, year | Country | n | Pre-HD Hb (g/dl) | Post-HD Hb (g/dl) | Intradialysis BW loss |
| --- | --- | --- | --- | --- | --- |
| Bellizzi et al, 2002 [3] | Italy | 49 | 11.0, SE0.1 | 12.6, SE0.2 | 3.4, SE0.1 |
|  |  |  | (95% CI, 10.7–11.3) | (95% CI, 12.2–13.0) | (95% CI, 3.1–3.6) |
| Movilli et al, 2002 [4] | Italy | 68 | 10.5 (1.3) | 11.5 (1.3) | Median, 3 kg |
|  |  |  |  |  | (range, 0.5 to 7 kg) |
| Korzets et al, 2002 [5] | Israel | 75 | 11.3 (1.6) | 12.8 (2.0) | Pre 69.7 (14.8), kg |
|  |  |  |  |  | Post 67.1 (14.3), kg |
| Minutolo et al, 2003 [6] | Italy | 32 | NOR, 14.4 (1.2) | NOR, 16.3 (1.9) | NOR, 4.0 (0.9), % |
|  |  |  | LOW, 11.4 (0.8) | LOW, 12.7 (0.9) | LOW, 4.1 (0.9), % |
| Geller et al, 2010 [7] | U.S. | 173 | 11.9 (1.4) | 12.8 (1.8) | 3.5 (1.6), % |
| Castillo et al, 2012 [8] | Spain | 67 | 11.7 (1.1) | 12.5 (1.2) | 2.19 ± 0.79 kg |
| Sagheb et al, 2016 [9] | Iran | 52 | 11.1 (1.1) | 11.9 (1.2) | 2.26 (0.89), kg |
| Nishiwaki et al, 2017 [10] | Japan | 230 | 10.8 (1.4) | 11.7 (1.7) | 4.4 (1.8), % |

Notation of each variable follows that of the referenced article. Each variable is presented with mean (SD). Hb, hemoglobin; HD, hemodialysis; BW, body weight; SE, standard error; CI, confidence interval; SD, standard deviation. In the study of Minutolo et al, NOR was defined as pre-dialysis Hb level 13 g/dl or more, and LOW was defined as a pre-dialysis Hb level of less than 13 g/dl.
