# Supplementary material for: The association of the difference in hemoglobin levels before and after hemodialysis with the risk of 1-year mortality in patients undergoing hemodialysis. Results from a nationwide cohort study of the Japanese Renal Data Registry
Source: PLoS One. 2019 Jan 10;14(1):e0210533. doi: 10.1371/journal.pone.0210533 (PMC6328160; doi:10.1371/journal.pone.0210533)
Supplement: S2 Table — (DOCX) [file pone.0210533.s006.docx]

S2 Table. Patient characteristics stratified by post-hemodialysis hemoglobin

|  | All | | | | Categorized by post-HD Hb | | | | | | | |
| --- | --- | --- | --- | --- | --- | --- | --- | --- | --- | --- | --- | --- |
|  | n = 34,187 | | Missing | | < 10 g/dl | | ≥ 10 to < 11 g/dl | | ≥ 11 to < 12 g/dl | | ≥ 12 g/dl | |
|  |  |  | n | (%) | n = 5,933 (18.9%) | | n = 7,494 (21.9%) | | n = 9,005 (25.8%) | | n = 11,755 (33.5%) | |
| Age, years (Q1, Q3) | 66 | (57, 74) | 0 | 0.0% | 69 | (60, 77) | 67 | (59, 75) | 66 | (58, 74) | 63 | (54, 72) |
| Sex, female (%) | 13,178 | (38.6%) | 0 | 0.0% | 2,529 | (42.6%) | 3,052 | (40.7%) | 3,533 | (39.2%) | 4,064 | (34.6%) |
| Median duration of dialysis, years (Q1, Q3) | 6 | (3,11) | 2 | 0.0% | 6 | (2, 10) | 6 | (3, 10) | 5 | (3, 10) | 6 | (3, 11) |
| Cause of ESRD, n (%) |  |  | 0 | 0.0% |  |  |  |  |  |  |  |  |
| Glomerulonephritis | 14,507 | (42.4%) |  |  | 2,336 | (39.4%) | 3,062 | (40.9%) | 3,834 | (42.6%) | 5,566 | (44.9%) |
| Diabetic nephropathy | 11,391 | (33.3%) |  |  | 2,110 | (35.6%) | 2,653 | (35.4%) | 3,053 | (33.9%) | 4,120 | (30.4%) |
| Nephrosclerosis | 2,200 | (6.4%) |  |  | 411 | (6.9%) | 495 | (6.6%) | 607 | (6.7%) | 797 | (5.8%) |
| PKD | 1,138 | (3.3%) |  |  | 207 | (3.5%) | 247 | (3.3%) | 273 | (3.0%) | 443 | (3.5%) |
| RPGN | 225 | (0.7%) |  |  | 36 | (0.6%) | 46 | (0.6%) | 56 | (0.7%) | 101 | (0.7%) |
| Others | 2,407 | (7.0%) |  |  | 386 | (6.5%) | 509 | (6.8%) | 611 | (6.8%) | 966 | (7.7%) |
| Unknown | 2,319 | (6.8%) |  |  | 447 | (7.5%) | 482 | (6.4%) | 571 | (6.3%) | 931 | (7.0%) |
| Vascular Access |  |  | 1,781 | 5.2% |  |  |  |  |  |  |  |  |
| AVF | 29,130 | (89.8%) |  |  | 4,918 | (87.4%) | 6,356 | (89.5%) | 7,707 | (90.3%) | 10,149 | (91.1%) |
| AVG | 2,420 | (7.5%) |  |  | 486 | (8.6%) | 551 | (7.8%) | 624 | (7.3%) | 759 | (6.8%) |
| Others | 856 | (2.6%) |  |  | 222 | (4.0%) | 197 | (2.8%) | 202 | (2.4%) | 235 | (2.1%) |
| Comorbidities |  |  |  |  |  |  |  |  |  |  |  |  |
| Cardiovascular disease (%) | 7,041 | (24.5%) | 5,458 | 16.0% | 1,434 | (28.3%) | 1,584 | (25.0%) | 1,758 | (23.4%) | 2,265 | (23.1%) |
| Amputation (%) | 857 | (3.0%) | 5,445 | 15.9% | 183 | (3.6%) | 173 | (2.7%) | 199 | (2.7%) | 302 | (3.1%) |

HD, hemodialysis; Hb, hemoglobin; Q1, first quartile; Q3, third quartile; ESRD, end-stage renal disease; PKD, polycystic kidney disease; RPGN, rapid progressive glomerulonephritis; AVF, arteriovenous fistula; AVG, arteriovenous graft. All categorical values are presented with n (%). Denominator of missing variables is the total number of patients (n = 38,636).
