# Supplementary material for: The association of the difference in hemoglobin levels before and after hemodialysis with the risk of 1-year mortality in patients undergoing hemodialysis. Results from a nationwide cohort study of the Japanese Renal Data Registry
Source: PLoS One. 2019 Jan 10;14(1):e0210533. doi: 10.1371/journal.pone.0210533 (PMC6328160; doi:10.1371/journal.pone.0210533)
Supplement: S3 Table — (DOCX) [file pone.0210533.s007.docx]

S3 Table: Laboratory data stratified by post-hemodialysis hemoglobin

|  | All | | | | Categorized by post-HD Hb | | | | | | | |
| --- | --- | --- | --- | --- | --- | --- | --- | --- | --- | --- | --- | --- |
|  | n = 34,187 | | Missing | | < 10 g/dl | | ≥ 10 to < 11 g/dl | | ≥ 11 to < 12 g/dl | | ≥ 12 g/dl | |
|  |  |  | n | % | n = 5,933 (18.9%) | | n = 7,494 (21.9%) | | n = 9,005 (25.8%) | | n = 11,755 (33.5%) | |
| Post-HD Hb, mean, g/dl | 11.3 | (10.3, 12.4) | 0 | 0.0% | 9.3 | (8.7, 9.7) | 10.5 | (10.2, 10.7) | 11.4 | (11.2, 11.7) | 12.8 | (12.3, 13.6) |
| Pre-HD Hb, mean, g/dl | 10.4 | (9.6, 11.1) | 0 | 0.0% | 8.9 | (8.3, 9.4) | 9.9 | (9.5, 10.3) | 10.5 | (10.0, 10.9) | 11.3 | (10.8, 12.0) |
| Post- and Pre-HD Hb difference, g/dl | 1.0 | (0.4, 1.5) | 0 | 0.0% | 0.3 | (-0.1, 0.6) | 0.6 | (0.2, 1.0) | 1.0 | (0.6, 1.4) | 1.6 | (1.1, 2.1) |
| Pre-HD serum albumin, mg/dl | 3.7 | (3.5, 4.0) | 611 | 1.8% | 3.6 | (3.3, 3.9) | 3.7 | (3.5, 4.0) | 3.8 | (3.5, 4.0) | 3.8 | (3.5, 4.0) |
| Pre-HD BUN, mg/dl | 64 | (54, 75) | 9 | 0.0% | 59 | (48, 70) | 62 | (52, 73) | 65 | (54, 75) | 68 | (57, 78) |
| Pre-HD serum creatinine, mg/dl | 10.5 | (8.6, 12.4) | 11 | 0.0% | 9.3 | (7.5, 11.0) | 10.0 | (8.4, 11.8) | 10.5 | (8.8, 12.4) | 11.4 | (9.6, 13.3) |
| Pre-HD sodium, mEq/L | 139 | (137, 141) | 21 | 0.1% | 139 | (137, 141) | 139 | (137, 141) | 139 | (137, 141) | 139 | (137, 141) |
| Pre-HD potassium, mEq/L | 5.0 | (4.4, 5.5) | 21 | 0.1% | 4.8 | (4.2, 5.3) | 4.9 | (4.4, 5.5) | 5.0 | (4.5, 5.5) | 5.1 | (4.6, 5.6) |
| Pre-HD calcium (adjusted), mg/dl | 9.3 | (8.8, 9.9) | 676 | 2.0% | 9.4 | (8.9, 10.0) | 9.3 | (8.8, 9.9) | 9.3 | (8.8, 9.8) | 9.3 | (8.8, 9.8) |
| Pre-HD phosphate, mg/dl | 5.2 | (4.3, 6.1) | 131 | 0.4% | 4.8 | (3.9, 5.8) | 5.0 | (4.2, 5.9) | 5.2 | (4.4, 6.1) | 5.5 | (4.6, 6.5) |
| CRP | 0.11 | (0.05, 0.38) | 4019 | 11.8% | 0.2 | (0.07, 0.74) | 0.11 | (0.05, 0.40) | 0.10 | (0.05, 0.30) | 0.10 | (0.05, 0.30) |
| Kt/V, ml/min | 1.39 | (1.22, 1.58) | 305 | 0.9% | 1.34 | (1.16, 1.55) | 1.38 | (1.19, 1.57) | 1.39 | (1.23, 1.59) | 1.41 | (1.25, 1.61) |
| nPCR, g | 0.87 | (0.76, 0.99) | 259 | 0.8% | 0.80 | (0.69, 0.93) | 0.85 | (0.73, 0.96) | 0.88 | (0.77, 1.00) | 0.92 | (0.80, 1.03) |
| BMI, kg/m^2^ | 20.8 | (18.8, 23.1) | 4555 | 13.3% | 20.2 | (18.3, 22.3) | 20.5 | (18.6, 22.8) | 20.9 | (18.9, 23.0) | 21.2 | (19.2, 23.6) |
| %ΔBW, % | 4.6 | (3.5, 5.7) | 206 | 0.5% | 4.1 | (2.8, 5.3) | 4.4 | (3.3, 5.5) | 4.6 | (3.6, 5.6) | 5.0 | (4.0, 6.0) |

Hb: hemoglobin; HD, hemodialysis; BUN: blood urea nitrogen; CRP, C-reactive protein; nPCR, normalized protein catabolic rate; BMI, body mass index; BW: body weight. All variables are presented as median (1st quartile, 3rd quartile). Denominator of missing variables is the number of all patients (n = 38,636).
