# Supplementary material for: The association of the difference in hemoglobin levels before and after hemodialysis with the risk of 1-year mortality in patients undergoing hemodialysis. Results from a nationwide cohort study of the Japanese Renal Data Registry
Source: PLoS One. 2019 Jan 10;14(1):e0210533. doi: 10.1371/journal.pone.0210533 (PMC6328160; doi:10.1371/journal.pone.0210533)
Supplement: S4 Table — (DOCX) [file pone.0210533.s008.docx]

S4 Table: Correlation coefficients of each candidate confounding variables

|  | Age | HD vintage | Times of HD sessions | Pre-HD albumin | Pre-HD BUN | Pre-HD creatinine | Pre-HD sodium | Pre-HD potassium | Pre-HD calcium (adjusted) | Pre-HD phosphate | Kt/V | PCR | BMI | %ΔBW | CRP |
| --- | --- | --- | --- | --- | --- | --- | --- | --- | --- | --- | --- | --- | --- | --- | --- |
| Age | 1 |  |  |  |  |  |  |  |  |  |  |  |  |  |  |
| HD vintage | –0.18 | 1 |  |  |  |  |  |  |  |  |  |  |  |  |  |
| Times of HD session | –0.27 | 0.31 | 1 |  |  |  |  |  |  |  |  |  |  |  |  |
| Pre-HD albumin | –0.32 | 0.03 | 0.15 | 1 |  |  |  |  |  |  |  |  |  |  |  |
| Pre-HD BUN | –0.16 | 0.07 | 0.09 | 0.27 | 1 |  |  |  |  |  |  |  |  |  |  |
| Pre-HD creatinine | –0.48 | 0.23 | 0.31 | 0.4 | 0.4 | 1 |  |  |  |  |  |  |  |  |  |
| Pre-HD sodium | –0.04 | 0.07 | 0 | 0.15 | –0.01 | 0.1 | 1 |  |  |  |  |  |  |  |  |
| Pre-HD potassium | –0.14 | 0.11 | 0.13 | 0.24 | 0.44 | 0.32 | –0.04 | 1 |  |  |  |  |  |  |  |
| Pre-HD calcium  (adjusted) | –0.03 | 0.22 | 0.08 | –0.22 | –0.07 | 0.03 | –0.04 | 0 | 1 |  |  |  |  |  |  |
| Pre-HD phosphate | –0.26 | 0.03 | 0.07 | 0.24 | 0.46 | 0.4 | 0.03 | 0.34 | –0.02 | 1 |  |  |  |  |  |
| Kt/V | –0.05 | 0.24 | 0.36 | 0.06 | 0.03 | 0.05 | 0 | 0.06 | 0.12 | –0.02 | 1 |  |  |  |  |
| nPCR | –0.1 | 0.11 | 0.15 | 0.16 | 0.5 | 0.23 | –0.01 | 0.26 | 0.01 | 0.23 | **0.70**^☨^ | 1 |  |  |  |
| BMI | –0.16 | –0.13 | 0.13 | 0.12 | 0.07 | 0.24 | 0.04 | 0.06 | –0.06 | 0.14 | –0.29 | –0.16 | 1 |  |  |
| %ΔBW | –0.01 | 0.02 | 0.02 | 0.02 | 0.01 | 0.03 | 0 | 0.04 | 0.02 | 0.02 | 0.63 | **0.85**^☨^ | –0.18 | 1 |  |
| CRP | 0.07 | –0.01 | –0.06 | –0.27 | –0.05 | –0.12 | –0.11 | –0.09 | 0.09 | -0.07 | –0.06 | –0.06 | –0.03 | –0.01 | 1 |

Hb: hemoglobin; HD, hemodialysis; ΔHb, difference in Hb levels before and after HD; BUN: blood urea nitrogen; nPCR, normalized protein catabolic rate; BMI, body mass index; BW: body weight; CRP, C-reactive protein
