# Supplementary material for: The association of the difference in hemoglobin levels before and after hemodialysis with the risk of 1-year mortality in patients undergoing hemodialysis. Results from a nationwide cohort study of the Japanese Renal Data Registry
Source: PLoS One. 2019 Jan 10;14(1):e0210533. doi: 10.1371/journal.pone.0210533 (PMC6328160; doi:10.1371/journal.pone.0210533)
Supplement: S5 Table — (DOCX) [file pone.0210533.s009.docx]

S5 Table. Patient characteristics stratified by measuring or not measuring post-hemodialysis hemoglobin

|  | Pre- and post-HD Hb measured | | | | Post-HD Hb not measured | | | |
| --- | --- | --- | --- | --- | --- | --- | --- | --- |
|  | n = 34,187 | | Missing | | n = 161,739 | | Missing | |
|  |  |  | n | (%) |  |  | n | (%) |
| Age, median years (1Q, 3Q) | 66 | (57, 74) | 0 | 0.0% | 66 | (58, 74) | 3 | 0.0% |
| Sex, female, n (%) | 13,178 | (38.6%) | 0 | 0.0% | 98,501 | (39.1%) | 0 | 0.0% |
| Duration of dialysis, years | 6 | (3,11) | 2 | 0.0% | 6 | (3,11) | 0 | 0.0% |
| Median years (1Q, 3Q) |  |  |  |  |  |  |  |  |
| Cause of ESRD, n (%) |  |  | 0 | 0.0% |  |  | 0 | 0.0% |
| Glomerulonephritis | 14,507 | (42.4%) |  |  | 70.754 | (43.8%) |  |  |
| Diabetic nephropathy | 11,391 | (33.3%) |  |  | 52,587 | (32.5%) |  |  |
| Nephrosclerosis | 2,200 | (6.4%) |  |  | 10,160 | (6.3%) |  |  |
| PKD | 1,138 | (3.3%) |  |  | 5,776 | (3.6%) |  |  |
| RPGN | 225 | (0.7%) |  |  | 943 | (0.6%) |  |  |
| Others | 2,407 | (7.0%) |  |  | 10,500 | (6.5%) |  |  |
| Unknown | 2,319 | (6.8%) |  |  | 11,019 | (6.8%) |  |  |
| Vascular access |  |  | 1,781 | 5.2% |  |  | 39,266 | 24.3% |
| AVF | 29,130 | (89.8%) |  |  | 109,944 | (89.7%) |  |  |
| AVG | 2,420 | (7.5%) |  |  | 8,986 | (7.1%) |  |  |
| Others | 856 | (2.6%) |  |  | 3,543 | (3.2%) |  |  |
| Comorbidities |  |  |  |  |  |  |  |  |
| Cardiovascular disease, % | 7,041 | (24.5%) | 5,458 | 16.0% | 27,178 | (19.9%) | 25,120 | 15.5% |
| Amputation, % | 857 | (3.0%) | 5,445 | 15.9% | 4,001 | (2.9%) | 24,832 | 15.4% |
| Mortality | 2,682 | (7.8%) | 0 | 0.0% | 12,644 | (7.8%) | 0 | 0.0% |

HD, hemodialysis; Hb, hemoglobin; 1Q, first quartile; 3Q, third quartile; ESRD, end-stage renal disease; PKD, polycystic kidney disease; RPGN, rapid progressive glomerulonephritis; AVF, arteriovenous fistula; AVG, arteriovenous graft; CVD, cardiovascular disease.
