# Supplementary material for: The association of the difference in hemoglobin levels before and after hemodialysis with the risk of 1-year mortality in patients undergoing hemodialysis. Results from a nationwide cohort study of the Japanese Renal Data Registry
Source: PLoS One. 2019 Jan 10;14(1):e0210533. doi: 10.1371/journal.pone.0210533 (PMC6328160; doi:10.1371/journal.pone.0210533)
Supplement: S6 Table — (DOCX) [file pone.0210533.s010.docx]

S6 Table: Laboratory data stratified by measuring or not measuring post-hemodialysis hemoglobin

|  | Pre- and post-HD Hb measured | | | | Post-HD Hb not measured | | | |
| --- | --- | --- | --- | --- | --- | --- | --- | --- |
|  | n = 34,187 | | Missing | | n = 161,739 | | Missing | |
|  |  |  | n | % |  |  | n | % |
| Post-HD Hb, g/dl | 11.3 | (10.3, 12.4) | 0 | 0.0% | NA | | NA | |
| Pre-HD Hb, g/dl | 10.4 | (9.6, 11.1) | 0 | 0.0% | 10.4 | (9.7, 11.2) | 0 | 0.0% |
| Pre-HD albumin, g/dl | 3.7 | (3.5, 4.0) | 611 | 1.8% | 3.7 | (3.5, 4.0) | 6,544 | 4.0% |
| Pre-HD BUN, mg/dl | 64 | (54, 75) | 9 | 0.0% | 65 | (54, 75) | 242 | 0.1% |
| Pre-HD serum creatinine, mg/dl | 10.5 | (8.6, 12.4) | 11 | 0.0% | 10.5 | (8.7, 12.5) | 188 | 0.1% |
| Pre-HD sodium, mEq/L | 139 | (137, 141) | 21 | 0.1% | 139 | (137, 141) | 1,500 | 0.9% |
| Pre-HD potassium, mEq/L | 5.0 | (4.4, 5.5) | 21 | 0.1% | 5.0 | (4.5, 5.5) | 220 | 0.1% |
| Pre-HD calcium (adjusted), mg/dl | 9.3 | (8.8, 9.9) | 676 | 2.0% | 9.3 | (8.8, 9.8) | 40,924 | 25.3% |
| Pre-HD phosphate, mg/dl | 5.2 | (4.3, 6.1) | 131 | 0.4% | 5.2 | (4.3, 6.1) | 374 | 0.2% |
| Protein catabolic rate | 0.11 | (0.05, 0.38) | 4,019 | 11.8% | 0.12 | (0.05, 0.39) | 41,075 | 25.4% |
| Kt/V, ml/min | 1.39 | (1.22, 1.58) | 305 | 0.9% | 1.39 | (1.22, 1.59) | 10,279 | 6.4% |
| nPCR, g | 0.87 | (0.76, 0.99) | 259 | 0.8% | 0.88 | (0.76, 1.00) | 10,061 | 6.2% |
| Body mass index, kg/m^2^ | 20.8 | (18.8, 23.1) | 4,555 | 13.3% | 20.7 | (18.7, 23.0) | 26,820 | 16.6% |
| %Δ body weight, % | 4.6 | (3.5, 5.7) | 206 | 0.5% | 4.6 | (3.5, 5.7) | 1,802 | 1.1% |

HD, hemodialysis; Hb, hemoglobin; NA, not available; BUN, blood urea nitrogen; nPCR, normalized protein catabolic rate.
